# Quality of Malaria Case Management in Malawi: Results from a Nationally Representative Health Facility Survey

**DOI:** 10.1371/journal.pone.0089050

**Published:** 2014-02-20

**Authors:** Laura C. Steinhardt, Jobiba Chinkhumba, Adam Wolkon, Madalitso Luka, Misheck Luhanga, John Sande, Jessica Oyugi, Doreen Ali, Don Mathanga, Jacek Skarbinski

**Affiliations:** 1 Malaria Branch, Division of Parasitic Diseases and Malaria, Centers for Disease Control and Prevention, Atlanta, Georgia, United States of America; 2 Malaria Alert Centre, College of Medicine, Blantyre, Malawi; 3 National Malaria Control Programme, Ministry of Health, Lilongwe, Malawi; 4 Malaria Branch, Division of Parasitic Diseases and Malaria, Centers for Disease Control and Prevention, Lilongwe, Malawi; Tulane University School of Public Health and Tropical Medicine, United States of America

## Abstract

**Background:**

Malaria is endemic throughout Malawi, but little is known about quality of malaria case management at publicly-funded health facilities, which are the major source of care for febrile patients.

**Methods:**

In April–May 2011, we conducted a nationwide, geographically-stratified health facility survey to assess the quality of outpatient malaria diagnosis and treatment. We enrolled patients presenting for care and conducted exit interviews and re-examinations, including reference blood smears. Moreover, we assessed health worker readiness (e.g., training, supervision) and health facility capacity (e.g. availability of diagnostics and antimalarials) to provide malaria case management. All analyses accounted for clustering and unequal selection probabilities. We also used survey weights to produce estimates of national caseloads.

**Results:**

At the 107 facilities surveyed, most of the 136 health workers interviewed (83%) had received training on malaria case management. However, only 24% of facilities had functional microscopy, 15% lacked a thermometer, and 19% did not have the first-line artemisinin-based combination therapy (ACT), artemether-lumefantrine, in stock. Of 2,019 participating patients, 34% had clinical malaria (measured fever or self-reported history of fever plus a positive reference blood smear). Only 67% (95% confidence interval (CI): 59%, 76%) of patients with malaria were correctly prescribed an ACT, primarily due to missed malaria diagnosis. Among patients without clinical malaria, 31% (95% CI: 24%, 39%) were prescribed an ACT. By our estimates, 1.5 million of the 4.4 million malaria patients seen in public facilities annually did not receive correct treatment, and 2.7 million patients without clinical malaria were inappropriately given an ACT.

**Conclusions:**

Malawi has a high burden of uncomplicated malaria but nearly one-third of all patients receive incorrect malaria treatment, including under- and over-treatment. To improve malaria case management, facilities must at minimum have basic case management tools, and health worker performance in diagnosing malaria must be improved.

## Background

Prompt and effective case management of uncomplicated *Plasmodium falciparum* malaria with artemisinin-based combination therapy (ACT) can reduce malaria mortality [1], as uncomplicated malaria can progress quickly to severe disease or death if not treated in time. Appropriate malaria case management relies on a complex, multiple-step process, from initial care-seeking to treatment adherence. The shift towards universal malaria diagnostic testing policies in many sub-Saharan African countries following the World Health Organization’s 2010 recommendation [2] has led to the scale-up of microscopy and malaria rapid diagnostic tests (RDTs), which should improve malaria diagnosis and treatment. However, health workers must still systematically identify patients with suspected malaria for testing, and must prescribe treatment and counsel patients appropriately. These activities have long been challenges for malaria case management in sub-Saharan Africa [3].

In 1993, in response to high rates of treatment failure, Malawi became the first country in sub-Saharan Africa to switch from chloroquine to sulfadoxine-pyrimethamine (SP) for treatment of uncomplicated malaria [4]. In 2007, due to growing resistance to SP, Malawi again changed its first-line antimalarial to the ACT, artemether-lumefantrine (AL). As of mid–2011, Malawi’s malaria case management policy still recommended presumptive treatment for children aged <5 years and for older patients if microscopy was not available; however, the Ministry of Health was planning to introduce rapid diagnostic tests (RDTs) later that year [2]. At the time, little was known about the quality of malaria case management at publicly-funded facilities, which are commonly used for treatment of fever and were reported to have managed nearly seven million suspected cases of malaria in 2010 based on routine National Malaria Control Programme data [5].

The objective of this study was to provide nationally representative estimates of the quality of malaria case management at publicly-funded health facilities in Malawi, using data from a nationally representative health facility survey conducted in April–May 2011. Specifically, we sought to assess the readiness of public facilities and health workers to provide malaria case management, characteristics of patients seeking care at public facilities, the proportion of patients with uncomplicated malaria who were treated correctly, and the proportion without clinical malaria who received malaria treatment (i.e., overtreatment).

## Methods

### Study Setting

Malaria is endemic throughout Malawi’s 28 districts, and all 15 million inhabitants are at risk for the disease. A 2010 nation-wide survey found a parasitemia prevalence of 43% among children aged <5 years [6], after several years of scaling up malaria control interventions [7]. Care at government-run health facilities is provided free of charge, and despite lower utilization rates among those living farther away from health centers [8], care-seeking for fever from health facilities is relatively high at 65% overall, and does not vary by wealth status [9]. Publicly funded health facilities in Malawi consist of government-run facilities and Christian Health Association of Malawi (CHAM) facilities, which are mission-run and charge a small fee to patients but receive some government funds. Health centers provide primary care services, and community hospitals, also called rural hospitals, provide both primary and secondary care. At the second level of the health system, district hospitals serve as referral facilities for health centers and community hospitals and provide primary care to their catchment area [10]. In 2011, Malawi’s latest malaria case management policy was from 2007 and recommended AL as the first-line antimalarial for uncomplicated malaria and artesunate-amodiaquine as second-line treatment in case of AL failure. Treatment for pregnant women in their first trimester and for children weighing <5 kilograms was oral quinine.

### Sampling and Survey Population

We conducted a three-stage, complex sample cross-sectional health facility survey. At the first stage, a stratified random sample of health facilities providing outpatient care was chosen from all Government of Malawi and CHAM health facilities; to help ensure geographic representativeness of results, four health facilities were selected in each of Malawi’s 28 districts. At the second stage, one outpatient department (OPD) per facility was selected (most facilities had only one). Within the selected OPD, a cluster was defined as all patient consultations performed on the day of the team’s visit during regular working hours (7∶30 am–5pm). At the third stage, a systematic sample of patients was chosen, with the sampling interval based on expected patient volume selected to yield approximately 20 eligible patients. Patients were eligible to participate if they were visiting the facility to see the health worker for the first time for their current illness and did not have signs of severe malaria (all patients) or any integrated management of childhood illness (IMCI) danger signs (children aged <5 years).

All eligible patients who consented to participate before seeing the health worker were given a study card on which the health workers recorded a unique health worker identification number, the results of any laboratory tests ordered, and all diagnoses given to the patient. Patients were interviewed by the study team after they had attended their consultation and collected any drugs prescribed. The study card and the patient’s health passport, which health workers use to document clinical care, were reviewed at the beginning of the patient exit interview. Surveyors administered a brief questionnaire about the patient’s symptoms, clinical encounter, and knowledge of how to take antimalarial medications, if prescribed. Patients had their temperature and weight measured and a thick and thin malaria blood smear prepared for later review. Surveyors performed a rapid diagnostic test (RDT) on all patients reporting fever or with a temperature ≥37.5°C who did not have a negative malaria diagnostic test result from the facility and who had not been prescribed an antimalarial; those testing positive were given a weight-appropriate AL dose. SD Bioline Malaria Pf® RDTs (Standard Diagnostics, Inc., Giheung-ku, Republic of Korea), which have been approved by the government of Malawi, were performed according to manufacturer recommendations.

In addition, all health workers providing clinical consultations in the selected OPD on the day of the visit were interviewed about their training, access to malaria guidelines, and supervision. Finally, a health facility assessment at each sampled facility was conducted through interviews of the facility in-charge and direct observation and collection of data on facility equipment, staffing, and infrastructure. After an eight-day training, teams of four surveyors, composed of three nurses and one clinical officer, carried out data collection from April 28–May 26, 2011 using Dell X5 personal digital assistants (PDAs) (Round Rock, TX) running Windows Mobile 5 (Redmond, WA) and programmed using Visual CE (Cambridge, MA).

### Laboratory Analysis

All blood slides were stained with Field’s stain A and B within seven days and read independently by two laboratory technicians at the Malaria Alert Centre in Blantyre. Asexual stage parasites were counted in thick smears against at least 200 white blood cells (WBCs), and parasite densities were calculated assuming 8,000 WBCs/dL of blood. A thick smear was considered positive if one or more malaria parasites was visualized and negative if no parasites were found after counting 500 fields. Both positive and negative discordant results, as well as results discordant on gametocyte presence or with >20% difference in parasite density, were re-read by a third expert laboratory technician from the Blantyre District Health Office whose readings were used as the final result.

### Outcome Definition and Statistical Analysis

Our primary outcome was correct treatment of uncomplicated clinical malaria, which was defined as parasitemia on the exit interview blood smear, plus either measured fever (temperature on re-examination ≥37.5°C) or a history of fever (defined as one or more of the following: 1) patient report during the exit interview that their illness involved a fever; 2) patient spontaneously mentioned fever complaint to health worker; 3) patient reported a symptom of fever to the surveyor when probed). Correct treatment was defined as prescription of an appropriate antimalarial (in most cases an ACT (AL or artesunate-amodiaquine) or oral quinine for those weighing <5 kg or for pregnant women in their first trimester) for patients with malaria. Overtreatment was defined as prescription of an ACT to patients without malaria, as measured by the exit interview blood smear.

The sample size was calculated to estimate the proportion of patients with uncomplicated malaria who received appropriate case management with a precision of ±10% in each of Malawi’s three regions, assuming: four facilities per district would be surveyed to ensure geographic representation; 20 outpatients would be surveyed per facility per day; of these, 5 (25%), would have clinical malaria; a prevalence of correct malaria treatment of 75% (based on case management surveys in neighboring countries [11], [12]); and a design effect of two, yielding a target sample size of 720 patients per region and 2,160 nationally. Analyses were also planned to compare case management of patients aged <5 years to older patients, as well as among the different facility types, although the study was not specifically powered to test for health facility-level differences.

Frequencies and cross-tabulations were calculated using the survey commands in Stata Version 11.0 (College Station, TX) to account for the complex survey design. Sample weights were calculated as the inverse of the probability of selection and thus all estimates are presented as weighted percentages or means and are regionally and nationally representative estimates. Key case management variables were tested for differences by patient age (<5 years vs. ≥5 years) using Rao-Scott chi-square tests.

### Estimation of Annual Number of Patients and Commodity Needs

Because all facilities and patients were sampled with a known probability of selection, and data were weighted as the inverse of the probability of selection, we used the sum of the weights to estimate the number of patients with uncomplicated malaria at public health facilities in Malawi, as well as RDT and ACT needs for effective case management. The probability of patient selection was calculated as (1/probability of facility selection * 1/number of OPDs at the facility * 1/sampling interval used). For most districts, the probability of facility selection equaled 4/number of facilities with OPDs in the district sampling frame, except in three districts. In two districts, one of the selected facilities did not have a functional OPD and the probability of selection became 3/(# facilities with OPDs in the district sampling frame –1). In the third district, Likoma, a survey team was able to visit only one of the two facilities in the district and the probability of selection of this facility was ½. The inverse of the selection probability was then multiplied by the inverse of the patient response rate for the health facility, equal to the number of patients completing the survey divided by the number of eligible patients. Since we had no information on the eligibility status of patients who refused the eligibility screening, we made the assumption that patients who refused would have been eligible in the same proportions as patients from whom we have eligibility data. To scale our estimates based on a survey conducted in April-May 2011 only and create annual estimates, we assumed that outpatient facilities are open five days per week for 50 weeks per year (accounting for weekday holidays), and that outpatient volume is constant throughout the year in Malawi. This extrapolation may overestimate the number of outpatients with malaria annually in Malawi, as the survey was conducted during the high transmission season, and annualization of malaria caseload was not adjusted for seasonality, given the lack of necessary data required to make this adjustment.

### Ethical Approval

Individual written informed consent was obtained from all eligible patients before conducting interviews. Written consent for children aged <7 years was obtained from the guardian or parent. For patients aged 7–17 years, assent was also obtained from the patient in addition to consent from the guardian or parent. In an effort to maintain confidentiality, participants’ data were linked to a unique identifier, and patient names were not recorded. Verbal consent was obtained from health workers before interviews and was documented in the PDA. The National Malaria Control Programme agreed to verbal consent for health workers, as questions were similar to those posed during routine supervision visits. The Malawi College of Medicine Ethical Committee (Blantyre, Malawi) and the US Centers for Disease Control and Prevention (Atlanta, GA, USA) reviewed and approved the protocol prior to data collection.

## Results

**Figure 1:**
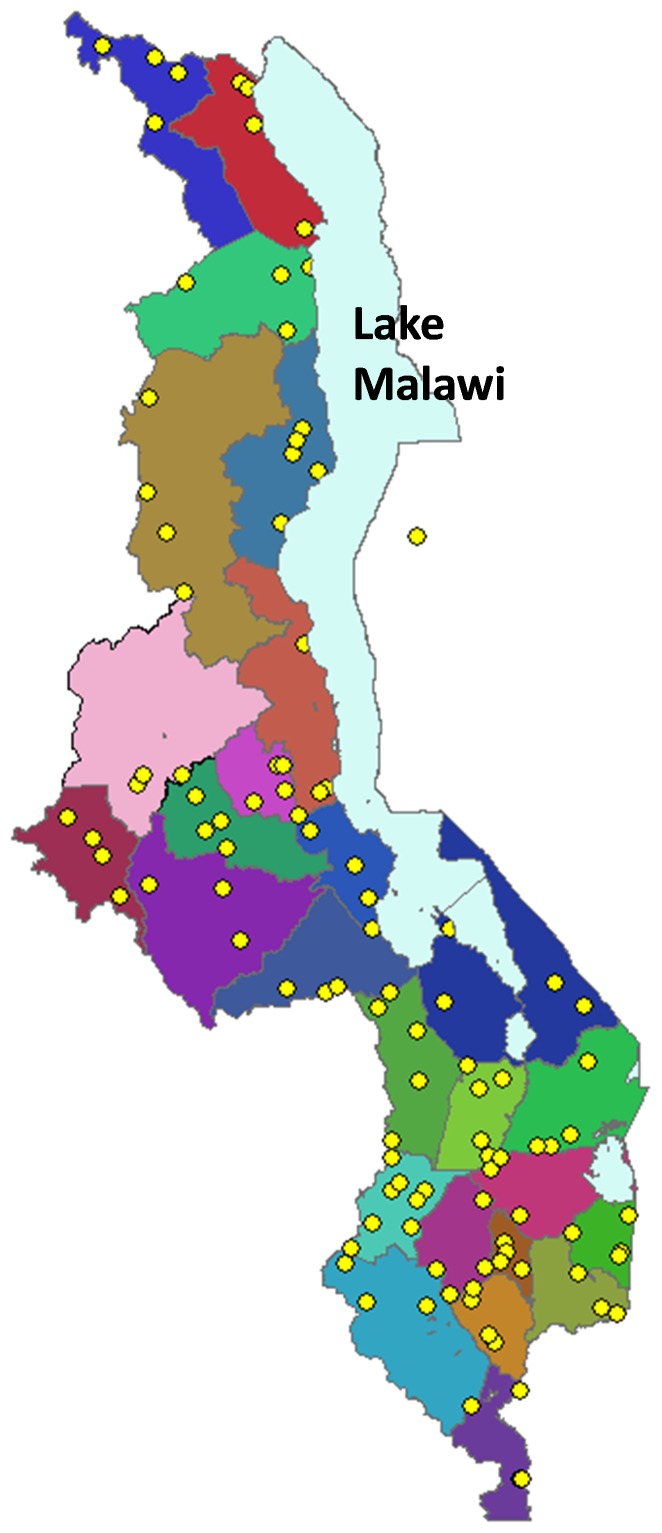
Map of surveyed health facilities. Note: Each yellow dot represents one surveyed facility.

A total of 107 health facilities, 136 health workers, and 2,019 patients with complete exit interview and blood smear data were surveyed across all 28 districts of Malawi (Table 1 and Figure 1). In two districts, only three of the four selected facilities were surveyed, as one did not have a functional OPD, and only one of the two facilities in Likoma district was surveyed for logistical reasons. Of 2,878 sampled patients: 2,630 (91%) consented to the eligibility screening; 2,260 (86%) were found to be eligible; and of these, 2,019 (89%) completed the exit interview. Assuming that patients who refused the eligibility screening were eligible at the same rate as those screened (86%), the overall patient response rate was 82%.

**Table 1:** Study sample.

	Region			Total
	North	Central	South	
Health facilities*	21	36	50	**107**
Health workers	25	45	66	**136**
Patients with complete interview and blood smear data	384	650	985	**2,019**

*Non-surveyed include those not visited for logistical reasons, (one in Likoma) or those visited but not functional (one in Balaka and another in Phalombe).

### Health Worker Characteristics

Health workers interviewed had an average age of 36.2 years and 10.6 years of clinical experience (Table 2). Most health workers (74.9%) were medical assistants, a cadre with two years of post-graduate training, although clinical officers, a position with three years of training, were also common in hospitals. Among all health workers, 82.5% had received some type of training on the 2007 malaria case management guidelines (through explicit malaria in-service training, on-the-job training, or IMCI training), and 67.5% had a written copy of the 2007 malaria treatment guidelines at the facility. Most health workers (82.5%) reported receiving at least one supervision visit in the previous six month, with those receiving supervision having a mean number of three visits in this time period; slightly more than half (58.6%) of these health workers reported that the supervision involved patient observation, and 39.7% reported that the supervision involved feedback.

**Table 2:** Demographics and training of health workers who provide outpatient care at publically-funded health facilities in Malawi, 2011.

	Type of Facility			Total N = 135%	p-value*
Characteristic	Health CentreN = 96%	District HospitalN = 16%	Rural HospitalN = 22%		
**Age, mean (range)**	36.2 (21, 77)	40.6 (22, 76)	33.2 (21, 68)	**36.2 (21, 77)**	0.129
**Female**	27.9	28.1	24.4	**27.3**	0.949
**Years of experience, mean (range)**	10.3 (0, 56)	15.2 (0, 42)	8.7 (0, 49)	**10.6** **(0, 56)**	0.170
**Facility in-charge**	70.7	0.0	9.6	**52.9**	<0.001
**Type of health worker**					0.002
Medical officer/doctor	0.6	0.0	4.5	**1.1**	
Medical assistant	76.8	75.6	65.6	**74.9**	
Clinical officer	4.4	24.4	30.0	**10.7**	
Nurse	18.2	0.0	0.0	**13.3**	
**Trained in:**					
Malaria case management (in-service training)	61.2	63.1	68.9	**62.6**	0.819
Malaria case management (on-the-job training)	37.9	49.7	49.4	**41.2**	0.528
Integrated management of childhood illness (IMCI)	65.1	55.2	50.8	**61.7**	0.460
Malaria case management after 2007, any method	84.4	72.9	80.5	**82.5**	0.506
**Has a copy of 2007 malaria treatment guidelines**	72.5	59.2	50.6	**67.5**	0.158
**Received any supervision in past 6 months**	88.4	70.7	64.2	**82.5**	0.040

Note: Differences assessed by chi-square statistics or t-tests, as appropriate, accounting for complex survey design.

### Facility Readiness

Forty-one percent of facilities surveyed had a microscope, and significantly fewer (24.4%) were able to perform microscopy on the day of the survey (Table 3). Few facilities were using RDTs, which had not yet been rolled out in Malawi. Hemoglobin testing capacity was low, with less than one-quarter of health facilities able to test hemoglobin levels. All district hospitals had malaria and anemia diagnostic testing capacity compared to 12% of health centers (Table 3). Where microscopy was available, the quality was low. Using reference blood smears from the exit interview as a gold standard, the sensitivity (46.1%; 95% confidence interval (CI) 36.9%, 57.1%) and specificity (75.2%; 95% CI 68.3%, 80.9%), of facility-based blood smears (N = 209) was low. A total of 81.4% of health centers had a functioning thermometer on the day of the survey. Most facilities (72.4%) had a copy of the most recent malaria treatment guidelines at the facility, and four health centers (3.9%) had a wall flowchart with the 2007 malaria treatment guidelines (Table 3).

**Table 3:** Availability of malaria diagnostics, infrastructure and equipment for malaria case management, malaria treatment guidelines, and antimalarials at publicly-funded outpatient health facilities in Malawi, 2011.

		Type of Facility				
		HealthCentreN = 86%	DistrictHospitalN = 8%	RuralHospitalN = 13%	p-value*	TotalN = 107%
**Diagnostic** **capacity**	**Microscopy**	28.9	100.0	93.3	<0.001	**40.6**
	**Microscopy functional for full day**	11.8	100.0	74.9	<0.001	**24.4**
	**Rapid diagnostic tests (RDTs)**	2.0	8.6	15.7	0.041	**4.0**
	**Hemoglobin (Hb) testing**	15.2	100.0	93.3	<0.001	**29.2**
	**Hb testing functional for full day**	12.0	100.0	78.4	<0.001	**24.9**
**Infrastructure** **and equipment**	**Electricity**	67.6	100.0	100.0	0.025	**73.3**
	**Water source**	79.8	100.0	100.0	0.137	**83.4**
	**Clean drinking water**	78.6	100.0	100.0	0.120	**82.4**
	**Cups/supplies for administering oral** **medications**	59.5	79.1	45.1	0.358	**59.1**
	**Functional hanging weighing scale**	93.2	87.8	92.2	0.859	**92.8**
	**Functional infant scale**	85.7	100.0	85.1	0.604	**86.6**
	**Functional standing scale**	79.2	100.0	85.1	0.415	**81.2**
	**Functional thermometer**	81.4	100.0	100.0	0.166	**84.7**
**Treatment** **guidelines**	**Copy of 2007 malaria treatment** **guidelines**	70.6	100.0	70.6	0.333	**72.4**
	**Wall flowchart with 2007 malaria** **treatment guidelines**	4.0	8.6	0.0	0.597	**3.9**
**ACT** **Stocks**	**Artemether-lumefantrine in stock** **for full day:**					
	1×6 (regular or dispersible)	69.8	85.6	92.2	0.193	**73.3**
	2×6 (regular or dispersible)	72.2	100.0	80.8	0.251	**74.9**
	3×6	64.7	100.0	76.9	0.145	**70.2**
	4×6	66.3	100.0	72.9	0.272	**69.1**
	Any AL dose-pack	77.3	100.0	100.0	0.097	**81.2**
	**Second-line treatment (artesunate-** **amodiaquine) ever stocked**	6.0	79.9	24.3	<0.001	**12.6**
**ACT** **stockouts**	**Mean number of days AL not in** **stock in past 3 months**					
	1×6 (regular or dispersible)	25.5	4.3	11.7	<0.001	**22.7**
	2×6 (regular or dispersible)	33.3	7.7	14.6	<0.001	**29.7**
	3×6	27.0	10.2	13.4	0.007	**24.5**
	4×6	21.6	5.0	7.6	<0.001	**19.0**

Differences assessed by chi-square statistics or t-tests, as appropriate, accounting for complex survey design.

Overall, 81.2% of facilities had at least one type of AL dose-pack in stock (Table 3). Since AL formulations are the same in all dose-packs, they can be split or combined to accommodate patients within different age/weight bands. However, stocks of the second-line antimalarial were limited to district hospitals (79.9% had artesunate-amodiaquine), and only 6.0% of community hospitals and health centers stocked artesunate-amodiaquine, p<0.001. Stock-outs of the different AL dose-packs in the three months prior to the survey were relatively common, ranging between an average of 19.0 and 29.7 days, and were significantly more common at health centers compared to hospitals, p<0.001. Oral quinine, which is used for treatment of pregnant women in their first trimester and children weighing <5 kg, was only available at 27.1% of health facilities. Availability of equipment for malaria treatment administration, such as clean water, cups, and weighing scales, was higher at district and rural hospitals than at health centers.

### Patient Case Management

**Figure 2:**
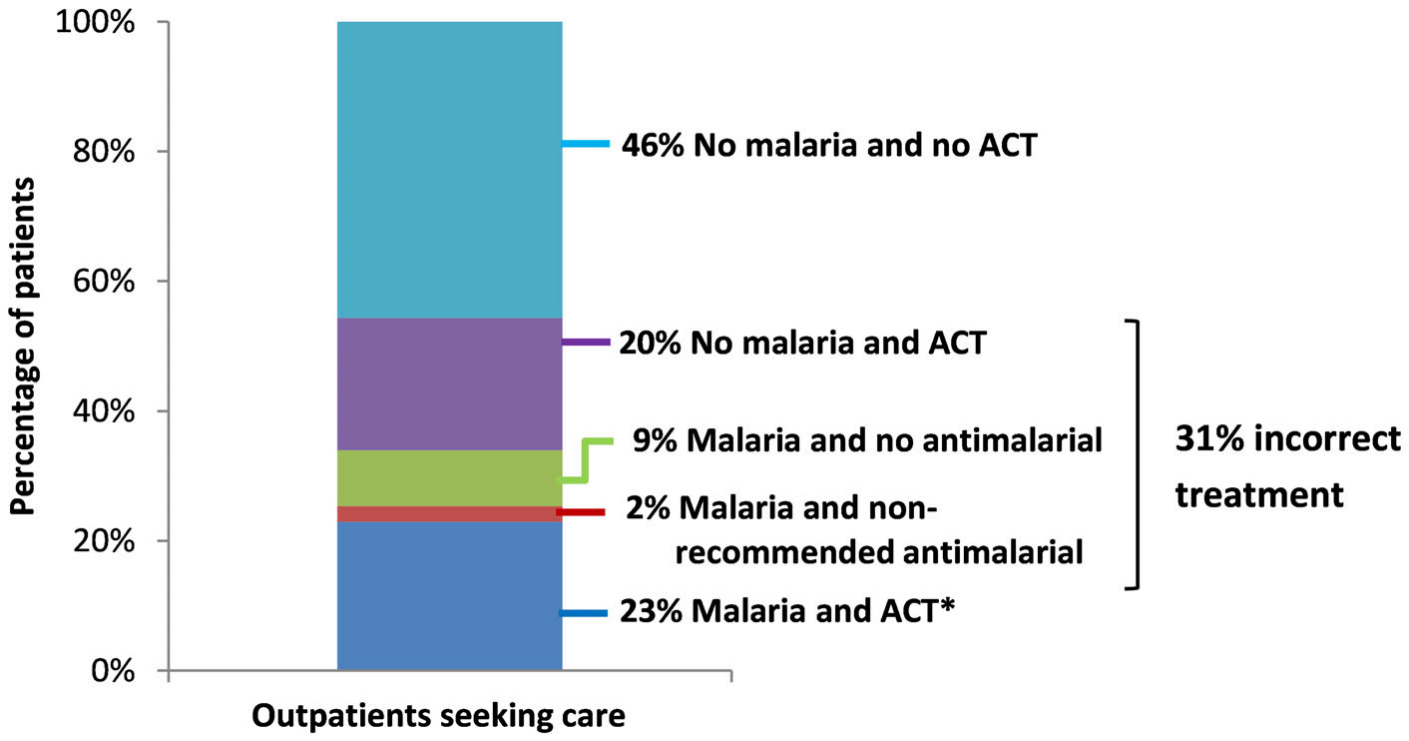
Malaria and case management among outpatients attending publicly-funded health facilities in Malawi (N = 2019). Note: Percentages are weighted. * Includes 1 pregnant patient in her first trimester who received oral quinine (correct treatment).

Among all outpatients, 34.0% had clinical malaria, defined as parasitemia on the exit interview blood smear and measured fever or history of fever (Table 4, Figure 2). Significantly more children aged <5 years had malaria (45.8%) compared to patients aged ≥5 years (28.7%), p<0.0001. Of all patients seeking curative care at outpatient departments, 31% received incorrect malaria treatment: 20% of patients without malaria were overtreated with an ACT and 11% of patients with clinical malaria received either no malaria treatment (9%) or a non-recommended antimalarial (2%) (Figure 2). Most patients (86.5%) presented with an illness involving a fever, and 27.8% had a temperature ≥37.5°C during the exit interview. Slightly more than half (55.1%) of patients said they spontaneously reported a complaint of fever to the health worker. Among patients who did not spontaneously report fever to the health worker, only 34.2% were asked by a health worker about the presence of fever and only 9.1% had their temperature measured (Table 4). Health workers did not assess fever by any means in 27.7% of all patients, and this was significantly more common in patients aged ≥5 years (39.0%) compared to those <5 years (9.2%), p<0.0001.

**Table 4:** Malaria diagnosis and treatment among outpatients attending publically-funded health facilities in Malawi, 2011.

Characteristic	Patient age			
	<5 years N = 806%	≥5 years N = 1,209%	p-value	Total N = 2,019%
Presented with an illness involving a fever*	94.1	81.8	<0.0001	**86.5**
High temperature (≥37.5°C) during exit interview	38.7	21.2	<0.0001	**27.8**
Positive reference blood smear (exit interview)	45.8	28.7	0.0012	**35.2**
Uncomplicated malaria (fever and positive reference blood smear)	45.5	27.0	0.0004	**34.0**
**Health worker assessment of fever**				
Fever spontaneously reported	79.7	40.1	<0.0001	**55.1**
Health worker asked patient about fever**	46.9	31.6	0.0277	**34.2**
Temperature taken**	19.5	7.0	0.0005	**9.1**
Temperature not asked or taken (and not reported by patient)	9.2	39.0	<0.0001	**27.7**
**Malaria diagnosis and treatment at facilities with microscopy**	*n = 234*	*n = 216*		***n = 450***
Blood smear (BS) performed	45.3	49.5	∧	**48.1**
BS performed if health worker noted fever¶	51.8	60.7	∧	**56.4**
ACT prescription if positive BS	(87.6)	(96.0)	∧	**92.4**
ACT prescription if negative BS	26.9	20.1	∧	**22.1**
**Malaria diagnosis and treatment at facilities without microscopy**	*n = 572*	*n = 993*		***n = 1,569***
ACT prescription if health worker noted fever¶	65.5	54.6	∧	**60.2**
Prescription of ACT if health worker did not note fever¶	23.3	19.3	∧	**20.1**
**Correct diagnosis and treatment of patients with malaria** **(according to exit interview BS)**	*n = 269*	*n = 358*		***n = 629***
ACT prescription †	72.7	61.5	0.066	**67.1**
Health worker diagnosis of malaria	79.8	66.6	0.031	**73.2**
**Overtreatment of patients without malaria**	*n = 537*	*n = 851*		***n = 1,390***
ACT prescription	43.8	24.9	0.0009	**30.9**

*Includes positive responses for: 1) patient says illness involved a fever, 2) patient spontaneously mentioned fever complaint to health worker, 3) patient reported a symptom of fever to surveyor when probed, or temperature on re-examination was > = 37.5°C.

**If patient does not spontaneously report to health worker.

¶ Spontaneously reported by patient to health worker, reported by patient when prompted, or temperature ≥37.5°C according to health worker’s recorded temperature.

† ACT refers to ACT (most patients) or oral quinine for pregnant women in their first trimester or patients weighing less than 5 kg.

Note: Numbers in parentheses are based on 25–49 unweighted cases.

∧ Chi-squared test with Rao-Scott correction unable to be performed to be performed due to stratum with single sampling unit.

Among patients seen at facilities with functional microscopy (450 of 2,019 patients, or 21.9%), only 48.1% were tested for malaria, and this was only slightly more common (56.4%) if health workers noted fever in patients (Table 4). Among patients with a positive facility blood smear, 92.4% were prescribed an ACT, but 22.1% of patients with a negative facility blood smear were also prescribed an ACT. Among patients at facilities without functional microscopy (1,569 of 2,019 patients, or 78.1%), 60.2% were prescribed an ACT if the health worker noted they had fever, and only 20.1% of patients without ascertained fever were prescribed an ACT (Table 4).

Only 67.1% of patients with clinical malaria received correct treatment (Table 4). Correct treatment was slightly higher among children aged <5 years with malaria (72.7%) compared to those aged ≥5 years (61.5%), p = 0.066. Failure by the health worker to diagnose malaria, whether presumptively or with laboratory confirmation, was the most common cause of incorrect treatment of patients with uncomplicated malaria; only 73.2% of patients with malaria were diagnosed with malaria by the health worker (Table 4). Two-thirds (66.0%) of all patients did not have clinical malaria (negative reference blood slide or absence of fever). Overtreatment was common (30.9% of patients without malaria) and varied significantly by age group (43.8% of children aged <5 years versus 24.9% of patients aged ≥5 years, p = 0.0009) (Table 4). Limiting the analysis to facilities with AL in stock for the full day did not substantially change these results. However, the difference by patient age in correct treatment and health worker diagnosis of malaria among patients with clinical malaria became more pronounced, with children aged <5 years significantly more likely than older patients with malaria to receive an ACT and be diagnosed with malaria at facilities with AL in stock for the full day (see Table S1).

Prescription of antimalarials other than ACTs was relatively uncommon. Among all non-pregnant patients prescribed an antimalarial (N = 980), 88.5% were given an ACT (all received AL); the remaining patients received SP (8.6%), oral quinine (1.0%), injectable quinine (1.0%), or both oral and injectable quinine (0.7%).

Among patients prescribed an ACT, 94.8% were given the correct ACT dose according to either age or weight (Table 5). In general, correct dosing according to either age or weight was better for patients at the lowest or highest ends of the age/weight spectrum, but worse for patients in the two middle age/weight groups. Nearly 80% of patients prescribed an ACT had it dispensed at the health facility (Table 5), but this varied dependent on whether the facility had the ACT in stock for the full day (93.4% if in stock for the full day versus 14.9% if out-of-stock). Only 13.2% of patients were directly observed taking the first ACT dose at the facility as recommended by malaria guidelines (Table 5). Almost all patients (95.4%) reported receiving counseling on how to take the drug at home, but fewer received specific counseling messages, such as taking the drug with food, what to do in case of vomiting, and completing all doses/tablets. Overall, 76.4% of patients or their caregivers were able to describe the correct dosing schedule for the ACT they received.

**Table 5:** Artemisinin-based combination therapy (ACT) dosing and counseling among outpatients prescribed an ACT attending publically-funded health facilities in Malawi, 2011 (N = 887).

Characteristic	Percent
**Correct ACT dose prescribed:**	
By age or weight	94.8
All age groups	84.8
<3 years	98.8
3–8 years	66.2
9–14 years	54.0
14+ years	96.9
All weight groups	88.5
5–14 kg	94.1
15–24 kg	77.6
25–34 kg	59.9
35+ kg	95.0
**ACT dispensed to patient**	79.8
**First dose of ACT given at facility**	13.2
**Health worker observed swallowing of first dose**	8.2
**Patient counseled on:** 1	
How to take drug at home	95.4
Taking drug with food, milk or milk-containing drink	30.8
What to do in case of vomiting drug	6.7
Completing all doses/tablets even if feel better	43.2
**Patient knows correct dosing of drug**	76.4

1 Only among patients who were dispensed the ACT.

### Population Estimates of Malaria Cases and Malaria Commodities Needed

Extrapolating from the percentage of patients surveyed with malaria, we estimated that 4.4 million patients (95% CI: 3.6M, 5.2M) with malaria were seen in outpatient departments of publicly funded facilities in 2011 and a corresponding number of ACTs were needed (Table 6). Assuming all patients with fever or history of fever need to be tested with an RDT under Malawi’s new case management policy, 11.2 million (95% CI: 8.9M, 13.6M) RDTs are needed annually, not including additional RDTs for buffer stocks, lost materials, etc. Table 6 also presents RDT needs by dose pack. With 67% of patients with malaria getting correct treatment, and 31% of patients without malaria treated unnecessarily, we estimate that 1.5 million patients with malaria did not receive correct treatment with an ACT in 2011, and 2.7 million patients without malaria inappropriately received ACTs.

**Table 6:** Estimated national annual outpatient caseloads and malaria commodity needs at publically-funded health facilities in Malawi, 2011.

Description	Age		Total		
	<5 years	≥5 years	%	Est. population size	95% confidence interval
***Estimated caseloads***					
Outpatients seeking curative care	4,934,084	8,062,165	100.0	12,996,250	(10,292,080, 15,700,420)
Febrile outpatients	4,643,089	6,590,864	86.4	11,236,350	(8,888,185, 13,584,515)
Uncomplicated malaria cases	2,242,750	2,174,558	34.0	4,422,300	(3,619,788, 5,224,813)
***Commodity needs***					
RDTs, all patients*	4,643,089	6,590,864	100.0	11,236,350	(8,888,185, 13,584,515)
AL courses, by patient weight**			100.0	4,422,300	(3,619,788, 5,224,813)
1×6 (5–14 kg)	1,854,400		42.4	1,854,400	(1,467,203, 2,241,597)
2×6 (15–24 kg)	1,249,550		28.6	1,249,550	(766,146, 1,732,955)
3×6 (25–34 kg)		181,125	4.1	181,125	(89,767, 272,483)
4×6 (35+ kg)		1,084,550	24.8	1,084,550	(521,780, 1,647,320)

Note: Estimates by age may not sum to total due to rounding. Estimates may overestimate the number of outpatients with malaria annually in Malawi, as the survey was conducted during the high transmission season, and annualization of malaria caseload was not adjusted for seasonality, given the lack of necessary data required to do this.

*Assuming one RDT needed for each febrile patient, not accounting for buffer stocks, etc.

**Actual number needed for RDTs and AL courses, assuming ACTs are used only for patients with malaria after they receive a diagnostic test and not accounting for buffer stocks, lost materials, expiring stocks, etc. Estimate is based on diagnosis of patients by expert microscopy, and use of RDTs at facilities may require additional ACTs, given the higher test positivity rate of RDTs compared to microscopy. In addition to ACTs, 68,705 courses of quinine are also needed for the 0.8% of malaria patients <5 kg and the0.4% who are pregnant women in their first trimester.

## Discussion

Correct malaria case management requires trained health workers, properly equipped and stocked health facilities, and a complex series of steps that health workers must correctly perform. We conducted a nationally representative health facility survey that evaluated health worker and facility readiness to provide quality malaria case management, assessed malaria diagnosis and treatment practices, and estimated numbers of malaria cases annually in Malawi. We found that 31% of all outpatients were incorrectly treated for malaria (11% undertreatment; 20% overtreatment), resulting in 1.5 million outpatients with clinical malaria who received no or incorrect malaria treatment and 2.7 million without malaria who were overtreated.

Most health workers have been trained in malaria case management, have a copy of the malaria treatment guidelines, and/or have received some supervision. However, capacity for malaria diagnosis was poor, with 24% of facilities with functional microscopy and 19% of health centers lacking a functional thermometer. Almost one of five facilities did not have any first-line ACT in stock for the full day on the day of the survey and various ACT dose packs were stocked out on average one-quarter of the time in the previous three months.

Malaria exerts a large burden on Malawi’s health system; one-third of patients seeking curative care at publicly-funded health facilities had uncomplicated clinical malaria. Only two-thirds of these patients with malaria were correctly treated with an ACT, and the most common reason for failure to treat a malaria case was missed diagnosis. Nearly one-third of patients without malaria received an ACT; thus of all ACTs prescribed in Malawi, 47% were prescribed to patients without malaria. Although malaria diagnosis requires access to parasitologic testing, health workers did poorly even in assessing patients for fever, a critical first step in malaria case management, regardless of whether or not diagnostics are available. Nearly 40% of patients aged ≥5 years were not assessed for fever, and the primary reason for incorrect treatment of malaria was missed diagnosis.

Even among patients found to have fever, only 56.4% were referred for microscopy at facilities with diagnostic capacity, and more than a fifth of patients with negative blood smears were prescribed an ACT. Failure to systematically test targeted patient groups, even at facilities with diagnostic testing available, has been found in other studies [11]–[14], which have also found high rates of antimalarial prescription for test-negative patients [12], [15]. One potential reason for failure to diagnostically test patients and for non-adherence to negative blood smear results is lack of health worker trust in microscopy results [16], [17]. This behavior might not be irrational in Malawi, given the low sensitivity and specificity of facility blood smears compared to expert microscopy that we found. Although microscopy has the potential to reduce overprescription of antimalarials, quality can be poor, even under research conditions [18]. Focusing on quality assurance and quality control for facility microscopy, especially at district hospitals, should be a priority for Malawi. The RDT rollout, which began in November 2011, has the potential to expand health workers’ capacity to diagnostically confirm suspected malaria. In addition to improving treatment of patients with malaria, RDTs might have an even larger impact on reducing the consumption of ACTs [19]–[22]. Moreover, RDTs might improve treatment of non-malarial febrile illnesses [23], which can be fatal if mistreated [17] and will play a larger role as the proportion of fevers caused by malaria continues to decline in sub-Saharan Africa [24]; in Malawi, considered to have high malaria transmission, the percentage of children aged <5 years with malaria parasitemia in household surveys decreased from 43% in 2010 to 28% in 2012 [6], [25].

Health workers rarely prescribed non-recommended antimalarials for treating malaria. In addition, health worker drug dosing and counseling were relatively good, with nearly 95% of patients receiving the correct AL dose for age or weight. Patient recall of the correct dosing schedule if they were prescribed AL was moderately high, with slightly more than three-quarters of patients (or caregivers) knowing when to take the drug. However, a previous study in Malawi using follow-up to patients’ homes found that overall adherence to AL was only 65%, but receiving the first dose at the facility significantly improved adherence [26]. As only 13% of patients received the first dose of AL at the facility in this study, health workers should be encouraged to adhere to guidelines and administer the first AL dose at the facility. However, 17% of facilities did not have clean drinking water and 41% lacked cups; these basic supplies must be available to enable administration of the first AL dose.

This study had several limitations. Most notably, health workers at surveyed facilities were aware that the survey team was conducting an evaluation of malaria case management, which may have positively influenced their performance (known as the “Hawthorne” effect) [27]–[29]; however, as described above many malaria case management practices were poor. Patient consultations were also not directly observed, and survey teams relied on patient exit interviews, which may be subject to recall bias, to capture information about the consultation. In addition, our estimates of annual malaria cases and commodity needs are likely overestimates as we were unable to adjust for malaria seasonality and the survey was conducted at the peak of the malaria transmission season.

By addressing case management gaps identified in this survey, including low availability of basic tools for malaria case management (thermometers, diagnostics, ACTs, water and drinking cups), lack of systematic assessment of fever in patients, low use of diagnostics and poor test quality, and poor adherence to negative test results, officials can begin to tackle the formidable burden that malaria poses at health facilities in Malawi. In addition to improving the supply chain for key commodities, strategies to improve health worker performance should focus on consistent diagnosis of malaria, including consistent assessment of fever in all patients and ordering diagnostic tests when indicated, in addition to prescribing according to test results. Unless these diagnostic practices are emphasized, the rollout of RDTs and other efforts to improve case management will not substantially reduce the millions of Malawians who receive incorrect malaria treatment each year.

## Supporting Information

Table S1 Malaria diagnosis and treatment among outpatients attending publically-funded health facilities in Malawi with AL in stock for the full day, 2011.
(DOCX)
