# Supplementary material for: Quality of Malaria Case Management in Malawi: Results from a Nationally Representative Health Facility Survey
Source: PLoS One. 2014 Feb 20;9(2):e89050. doi: 10.1371/journal.pone.0089050 (PMC3930691; doi:10.1371/journal.pone.0089050)
Supplement: Table S1 — Malaria diagnosis and treatment among outpatients attending publically-funded health facilities in Malawi with AL in stock for the full day, 2011. (DOCX) [file pone.0089050.s001.docx]

| **Table S1: Malaria diagnosis and treatment among outpatients attending publically-funded health facilities in Malawi with AL in stock for the full day, 2011** | | | | |
| --- | --- | --- | --- | --- |
| **Characteristic** | **Patient age** | | |  |
|  | <5 years  N=630  % | ≥5 years  N=937  % | p-value | **Total**  **N=1,567**  **%** |
| Presented with an illness involving a fever* | 94.1 | 81.3 | <0.0001 | **86.0** |
| High temperature (≥37.5 °C) during exit interview | 38.2 | 19.9 | 0.0001 | **26.7** |
| Positive reference blood smear (exit interview) | 46.1 | 27.8 | 0.0032 | **34.5** |
| Uncomplicated malaria (fever and positive reference blood smear) | 45.7 | 26.2 | 0.0016 | **33.4** |
| **Health worker assessment of fever** |  |  |  |  |
| Fever spontaneously reported | 78.6 | 38.3 | <0.0001 | **53.2** |
| Health worker asked patient about fever** | 48.1 | 32.4 | 0.0490 | **35.0** |
| Temperature taken** | 18.7 | 6.5 | 0.0015 | **8.6** |
| Temperature not asked or taken (and not  reported by patient) | 9.4 | 39.8 | <0.0001 | **28.6** |
| **Malaria diagnosis and treatment at facilities with microscopy** | *n=171* | *n=216* |  | ***n=387*** |
| Blood smear (BS) performed | 44.4 | 49.7 | ⌃ | **48.0** |
| BS performed if health worker noted fever^¶^ | 50.3 | 60.7 | ⌃ | **56.2** |
| ACT prescription if positive BS | (95.6) | (97.5) | ⌃ | **96.8** |
| ACT prescription if negative BS | 27.4 | 20.0 | ⌃ | **22.0** |
| **Malaria diagnosis and treatment at facilities without microscopy** | *n=459* | *n=721* |  | ***n=1,180*** |
| ACT prescription if health worker noted  fever^¶^ | 69.3 | 53.8 | ⌃ | **61.9** |
| Prescription of ACT if health worker did not  note fever^¶^ | 25.9 | 15.6 | ⌃ | **17.1** |
| **Correct diagnosis and treatment of patients with malaria (according to exit interview BS)** | *n=269* | *n=358* |  | ***n=629*** |
| ACT prescription ^†^ | 75.8 | 58.2 | 0.0199 | **67.1** |
| Health worker diagnosis of malaria | 79.5 | 61.8 | 0.0102 | **70.8** |
| **Overtreatment of patients without malaria** | *n=349* | *n=733* |  | ***n=1,082*** |
| ACT prescription | 45.9 | 23.6 | 0.0003 | **30.4** |
| * Includes positive responses for: 1) patient says illness involved a fever, 2) patient spontaneously mentioned fever complaint to health worker, 3) patient reported a symptom of fever to surveyor when probed, or temperature on re-examination was >=37.5 C. | | | | |
| ** If patient does not spontaneously report to health worker. | | | | |
| ^¶^ Spontaneously reported by patient to health worker, reported by patient when prompted, or temperature >37.5° C according to health worker's recorded temperature. | | | | |
| ^†^ACT refers to ACT (most patients) or oral quinine for pregnant women in their first trimester or patients weighing less than 5 kg. | | | | |
| Note: Numbers in parentheses are based on 25-49 unweighted cases. | | | | |
| ⌃ Chi-squared test with Rao-Scott correction unable to be performed to be performed due to stratum with single sampling unit. | | | | |
